# Metabolomics and ultra-processed foods intake: A scoping review protocol

**DOI:** 10.1016/j.mex.2026.103974

**Published:** 2026-05-25

**Authors:** Talita do Nascimento Peixoto, Anna Luisa Moura Alencar Rocha, Lucia Fátima Campos Pedrosa

**Affiliations:** a Graduate Program in Health Sciences, Federal University of Rio Grande do Norte, Av. Cordeiro de Farias s/n, Natal 59012-570, RN, Brazil; b Graduate Program in Nutrition, Federal University of Rio Grande do Norte, Av. Salgado Filho, 3000-Lagoa Nova, Natal 59078-970, RN, Brazil; c Department of Nutrition, Federal University of Rio Grande do Norte, Av. Salgado Filho, 3000 - Lagoa Nova, Natal, RN 59078 970, Brazil

**Keywords:** Metabolomics, Ultra-processed foods, NOVA classification, Nutritional epidemiology

## Abstract

The association between the consumption of ultra-processed foods (UPFs) and adverse health outcomes remains unclear regarding its mechanistic explanation. In this context, metabolomics emerges as a promising approach for identifying biomarkers and understanding the metabolic effects of these foods on the body. However, the evidence linking metabolomic profiles to UPFs consumption remains limited and heterogeneous. This scoping review aims to map and synthesize the available scientific evidence on the use of metabolomics in the study of UPFs consumption, characterize the study designs and methodological approaches employed, and identify knowledge gaps. The review will be conducted according to the JBI methodology and reported in accordance with the PRISMA-ScR checklist. Searches will be performed in the PubMed/MEDLINE, Embase, LILACS and Web of Science databases. Two reviewers will independently screen titles, abstracts, and full-text articles for inclusion. Data will be extracted using a standardized form and synthesized narratively. This review is expected to provide a comprehensive overview of the available evidence, highlight methodological heterogeneity, and identify gaps that may guide future research. The protocol was registered in the Open Science Framework (OSF).

## Graphical abstract

**Figure:**
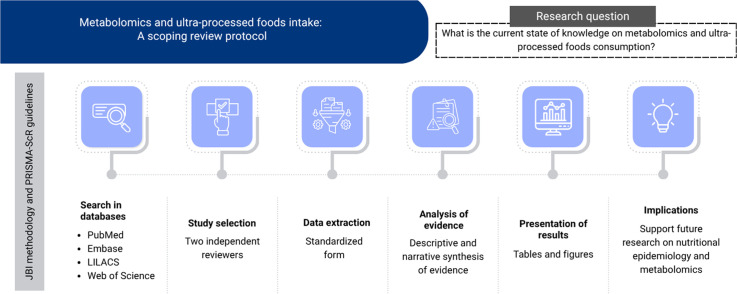

## Specifications table

**Table:** 

**Subject area**	Food Science
**More specific subject area**	Nutrition
**Name of your protocol**	Metabolomics and ultra-processed foods intake: A scoping review protocol
**Reagents/tools**	Not applicable
**Experimental design**	Not applicable. This protocol describes a scoping review
**Trial registration**	Not applicable. This scoping review is registered in the Open Science Framework (10.17605/OSF.IO/3YDWA)
**Ethics**	Ethical approval was not required, as this study is based on secondary data does not involve direct human or animal participation.
**Value of the Protocol**	1. Maps current evidence on the relationship between metabolomics and consumption of ultra-processed foods 2. Identifies methodological approaches, gaps, and inconsistencies in literature. 3. Supports future research and advances in nutritional epidemiology using omics-based approaches.

## Background

In the late 2000s, policymakers, researchers, and health professionals became interested in understanding food processing as a determinant of contemporary dietary patterns and its contribution to the rise in obesity and other chronic diseases. Until then, nutritional assessment systems and dietary recommendations focused mainly on food groups and their intrinsic relationships with energy and nutrient composition, placing little emphasis on the industrial processing stages of food [1,2].

In this context, global changes in food systems, characterized by the increasing availability of various ready-to-eat products, reinforced the need for approaches that incorporate food processing as a central element of dietary assessment. From this perspective, the NOVA classification was conceived as a tool to describe food systems and patterns and how they can affect health and disease risk. This classification categorizes foods into four groups according to the degree and purpose of processing: (1) fresh or minimally processed foods, (2) processed culinary ingredients, (3) processed foods, and (4) ultra-processed foods (UPF) [3].

UPFs are industrially produced formulations made primarily from isolated or modified food components that contain little or no whole food. In general, UPFs contain additives and are attractive because they are convenient, shelf-stable, and highly palatable [4]. In recent decades, consumption of these foods has increased significantly in many countries, raising concerns about public health and now accounts for a substantial portion of total energy intake, especially among children and adolescents. This scenario highlights the consolidation of an increasingly industrialized and globalized dietary pattern [5,6].

Scientific evidence links UPFs consumption to more than thirty adverse health outcomes, including obesity, cardiovascular disease, type 2 diabetes, and all-cause mortality [7,8]. However, many of these associations are based on self-reported measures of food intake, which are subject to memory and measurement biases [9], while the underlying biological mechanisms related to these diseases remain poorly understood. Additionally, factors such as nutritional imbalances, low intake of phytochemicals, and constant exposure to additives or contaminants from food processing can contribute to metabolic and inflammatory changes [10,11].

Therefore, nutritional epidemiology has incorporated omics-based approaches to overcome the limitations of traditional methods and elucidate molecular mechanisms [12]. Among these approaches, metabolomics stands out by characterizing the metabolome—the set of low-molecular-weight metabolites present in biological matrices such as blood, urine, and saliva. The identification of these molecules relies on advanced analytical platforms, such as nuclear magnetic resonance spectroscopy and chromatography coupled with mass spectrometry [13].

In the nutrition area, metabolomics has been used to identify dietary biomarkers, assess metabolic responses to specific dietary patterns, and examine the relationship between diet and health outcomes [[14], [15], [16]]. Some studies have explored associations between UPFs consumption and metabolomic profiles using different epidemiological designs, as well as various biological matrices and analytical platforms [[17], [18], [19], [20], [21]]. This diversity hinders comparisons of results and identifies consistent patterns in the metabolomic profiles associated with UPFs consumption.

Based on these aspects, this scoping review was designed to map available evidence on the topic using a systematic methodology guided by a broad research question [22].

## Description of protocol

This review will use the JBI [23] methodology and the PRISMA-ScR extension [24]. The protocol was registered on the OSF [25] under the DOI 10.17605/OSF.IO/3YDWA. To check for previous reviews, a preliminary search was conducted on April 1, 2026, across JBI Evidence Synthesis, the Cochrane Database of Systematic Reviews, PubMed, and PROSPERO. No reviews or protocols focused on metabolomics studies and UPFs consumption were found, reinforcing this proposal's relevance.

This study seeks to answer the following main question: What is the current state of knowledge regarding studies on metabolomics and UPFs consumption? Additionally, the following secondary questions will be addressed: (a) which analytical platforms and metabolomic approaches (targeted vs. untargeted) have been most commonly used; (b) which biological matrices have been analyzed; (c) which metabolite profiles or metabolomic signatures have been associated with UPFs consumption; and (d) in which countries and population contexts have the studies been conducted?

## Eligibility criteria

The eligibility criteria for this scoping review were defined using the Population, Concept, and Context (PCC) framework to ensure alignment with the research question. Primary studies conducted in humans that assess metabolomic characteristics associated with UPFs consumption will be included, with no restrictions on study design, context, or the biological matrix analyzed. For conceptual consistency, only studies employing high-throughput metabolomic platforms will be considered; studies assessing only isolated biomarkers, without a comprehensive metabolomic approach, will be excluded. Dietary exposure assessment must follow the NOVA classification [4]. Studies published in peer-reviewed journals will be included, while gray literature and non-peer-reviewed materials will be excluded to maintain methodological consistency. The inclusion and exclusion criteria are summarized in Table 1.

**Table 1:** Eligibility criteria for the scoping review.

Aspect	Inclusion	Exclusion
Population	Studies conducted with humans of any age group or health condition.	Studies using animal, cellular, or in vitro models.
Concept	Studies evaluating metabolomic characteristics associated with UPFs consumption.	Studies that assess only clinical outcomes or risk factors related to UPFs use without metabolomic analysis.
Context	Open – No geographical restrictions.	N/A
Time	Studies published through Jun 2026.	N/A
Literature	Primary studies published as original articles.	Gray literature (theses, dissertations, technical reports), conference abstracts without full text, editorials, letters to the editor, book chapters, and narrative and systematic reviews.
Study design	Observational and experimental studies, regardless of study design (e.g., cross-sectional, cohort, case-control, clinical trials)	Case reports and case series.
Assessment of dietary intake	Studies that assess dietary intake using the NOVA classification.	Studies that assess broad dietary patterns or individual foods without the ability to identify them as ultra-processed.
Metabolomic approaches	Studies that utilize targeted or untargeted approaches and applied analytical techniques.	Studies that rely solely on isolated biomarkers without a comprehensive metabolomic approach.

UPFs, ultra-processed foods; N/A, not applicable.

## Search strategy

The search will use PubMed/MEDLINE, Embase, LILACS, and Web of Science, ISI, combining Medical Subject Headings (MeSH) and free-text terms, as shown in Table 2. Queries will be adapted for each database. A preliminary search in PubMed/MEDLINE and Embase identified relevant studies and refined the strategy; keywords and indexing terms from these articles informed the final approach.

**Table 2:** Example of a search strategy for the database.

Date	Strategy	Database	Search results
April 8, 2026	("Food, Processed"[Mesh] **OR** "Ultra processed food" **OR** "Ultra-processed foods" **OR** "Processed foods" **OR** "NOVA classification" **OR** "NOVA food groups") **AND** (Metabolomics [Mesh] **OR** Metabolome [Mesh] **OR** Metabolomics **OR** Metabolomic **OR** Metabolites **OR** "Metabolite patterns")	PubMed	213

## Selection of studies

The identified studies will be entered into the Rayyan application [26], where duplicates will be removed, and an initial screening of titles and abstracts will be conducted. A pilot phase will be conducted beforehand to ensure consistency and alignment among reviewers in applying the eligibility criteria.

**Fig. 1:**
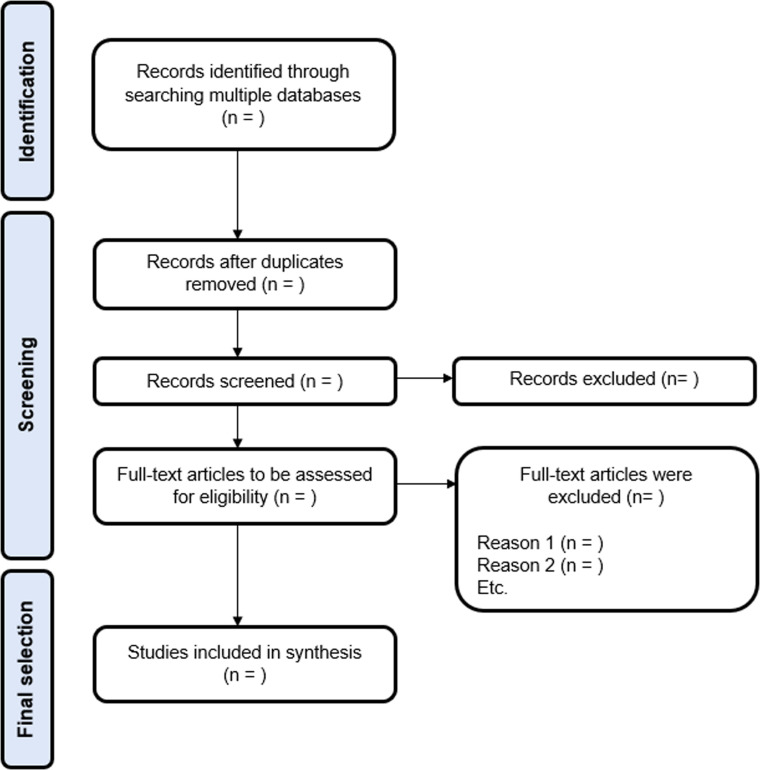
Example of a PRISMA-ScR flowchart.

The screening will be conducted independently by two reviewers based on the previously established inclusion criteria. Any disagreements will be resolved by consensus or, when necessary, by a third reviewer. Subsequently, potentially eligible studies will be evaluated in detail, and the reasons for exclusion will be duly recorded. A flowchart of the study selection process will be presented in accordance with the PRISMA-ScR statement [21] (Fig. 1).

## Data extraction

Data extraction will be performed independently by two reviewers using a form previously developed by the authors (Table 3). The instrument will be tested in advance for suitability and adjusted if needed during the review. Reviewer discrepancies will be resolved by consensus or, if necessary, by consulting a third reviewer. If information is incomplete or missing, study authors may be contacted for clarification.

**Table 3:** Data extraction.

Category	Data
Bibliographic information	Author(s)
	Title
	Year of publication
	Country where the study was conducted
	Journal or publication source
	Digital Object Identifier (DOI)
Study characteristics	Objective(s)
	Study Design
	Context/Population
	Sample Size
Participant Information	Gender
	Age
	Health status
Assessment of UPFs consumption	Method of dietary measurement and assessment
Metabolomic analysis	Biological matrix
	Analytical platform
	Approach (targeted or untargeted)
	Statistical methods
Key Results	Summary of key findings
	Significant associations
Key Findings	Discussion of key findings
	Reported limitations and strengths
Observations	Any additional information specific to the research question or objectives

UPFs, ultra-processed foods.

## Analysis of the evidence

The evidence will be presented descriptively, in accordance with the JBI recommendations for scoping reviews. The data will be organized and presented using simple descriptive statistics, including absolute and relative frequencies of the main characteristics of the studies, such as study design, population, methods for assessing food intake, and metabolomic approaches used.

In addition, a descriptive synthesis of the findings will be conducted to identify the main approaches, knowledge gaps, and patterns found in the literature. No quantitative synthesis of the results or assessment of methodological quality for the purpose of drawing inferences will be performed, given the exploratory nature of this scoping review.

## Presentation of results

The results will be presented using tables and figures to facilitate the visualization and understanding of the data, accompanied by a descriptive narrative summary. The study will also highlight key knowledge gaps, discuss the applicability of metabolomics to understanding the mechanisms involved in pathophysiological disorders related to diet quality, and outline implications for future research.

## Protocol validation

Not applicable.

## Related research article

None.

## Limitations

Not applicable.

## CRediT author statement

**Talita do Nascimento Peixoto**: Conceptualization, Methodology, Data curation, Formal analysis, Writing – Original Draft, Writing – Review & Editing; **Anna Luisa Moura Alencar Rocha:** Conceptualization, Methodology, Investigation, Writing – Review & Editing; **Lucia Fátima Campos Pedrosa:** Conceptualization, Methodology, Project administration, Funding acquisition, Supervision, Writing – Original Draft, Writing – Review & Editing.

## Declaration of competing interest

The authors declare that they have no known competing financial interests or personal relationships that could have appeared to influence the work reported in this paper.

## Data Availability

Data will be made available on request.
